# A genome-wide association study on medulloblastoma

**DOI:** 10.1007/s11060-020-03424-9

**Published:** 2020-02-13

**Authors:** Anna M. Dahlin, Carl Wibom, Ulrika Andersson, Jonas Bybjerg-Grauholm, Isabelle Deltour, David M. Hougaard, Michael E. Scheurer, Ching C. Lau, Roberta McKean-Cowdin, Rebekah J. Kennedy, Long T. Hung, Janis Yee, Ashley S. Margol, Jessica Barrington-Trimis, W. James Gauderman, Maria Feychting, Joachim Schüz, Martin Röösli, Kristina Kjaerheim, The Cefalo Study Group, Michaela Prochazka, Maral Adel Fahmideh, Birgitta Lannering, Lisbeth S. Schmidt, Christoffer Johansen, Astrid Sehested, Claudia Kuehni, Michael Grotzer, Tore Tynes, Tone Eggen, Lars Klæboe, Danuta Januszkiewicz-Lewandowska, Marta Fichna, Jerzy Nowak, Susan Searles Nielsen, Shahab Asgharzadeh, Lisa Mirabello, Ulf Hjalmars, Beatrice Melin

**Affiliations:** 1 Department of Radiation Sciences, Oncology, Umeå University, Umeå, Sweden; 2 Danish Center for Neonatal Screening, Department for Congenital Disorders, Statens Serum Institut, Copenhagen, Denmark; 3 Section of Environment and Radiation, International Agency for Research on Cancer, Lyon, France; 4 Unit of Statistics, Bioinformatics and Registry, Danish Cancer Society Research Center, Copenhagen, Denmark; 5 Department of Pediatrics, Section of Hematology-Oncology, Texas Children’s Cancer Center, Baylor College of Medicine, Houston, TX USA; 6 Department of Preventive Medicine, Keck School of Medicine, University of Southern California, Los Angeles, CA USA; 7 Children’s Center for Cancer and Blood Diseases, Children’s Hospital Los Angeles, Los Angeles, CA USA; 8 Department of Pediatrics, Section of Hematology-Oncology, Children’s Hospital Los Angeles and The Saban Research Institute, Keck School of Medicine of University of Southern California, Los Angeles, CA USA; 9 Unit of Epidemiology, Institute of Environmental Medicine, Karolinska Institutet, Stockholm, Sweden; 10 Department of Epidemiology and Public Health, Swiss Tropical and Public Health Institute, Basel, Switzerland; 11 University of Basel, Basel, Switzerland; 12 The Cancer Registry of Norway, Oslo, Norway; 13 Institute of Human Genetics, Polish Academy of Sciences, Poznan, Poland; 14 Department of Pediatric Oncology, Hematology and Bone Marrow Transplantation, Poznan University of Medical Sciences, Poznan, Poland; 15 Department of Endocrinology, Metabolism and Internal Medicine, Poznan University of Medical Sciences, Poznan, Poland; 16 Public Health Sciences Division, Fred Hutchinson Cancer Research Center, Seattle, WA USA; 17 Department of Neurology, School of Medicine, University of Washington, Seattle, WA USA; 18 Department of Pathology, Saban Research Institute at Children’s Hospital Los Angeles, Keck School of Medicine, University of Southern California, Los Angeles, CA USA; 19 Division of Cancer Epidemiology and Genetics, National Cancer Institute, National Institutes of Health, Bethesda, MD USA

**Keywords:** Pediatric cancers, CNS cancers, Adolescents and young adults (AYA), Epidemiology, Genetics of risk, outcome, and prevention

## Abstract

**Introduction:**

Medulloblastoma is a malignant embryonal tumor of the cerebellum that occurs predominantly in children. To find germline genetic variants associated with medulloblastoma risk, we conducted a genome-wide association study (GWAS) including 244 medulloblastoma cases and 247 control subjects from Sweden and Denmark.

**Methods:**

Genotyping was performed using Illumina BeadChips, and untyped variants were imputed using IMPUTE2.

**Results:**

Fifty-nine variants in 11 loci were associated with increased medulloblastoma risk (p < 1 × 10^–5^), but none were statistically significant after adjusting for multiple testing (p < 5 × 10^–8^). Thirteen of these variants were genotyped, whereas 46 were imputed. Genotyped variants were further investigated in a validation study comprising 249 medulloblastoma cases and 629 control subjects. In the validation study, rs78021424 (18p11.23, *PTPRM*) was associated with medulloblastoma risk with OR in the same direction as in the discovery cohort (OR_T_ = 1.59, p_validation_ = 0.02). We also selected seven medulloblastoma predisposition genes for investigation using a candidate gene approach: *APC*, *BRCA2*, *PALB2*, *PTCH1*, *SUFU*, *TP53*, and *GPR161*. The strongest evidence for association was found for rs201458864 (*PALB2*, OR_T_ = 3.76, p = 3.2 × 10^–4^) and rs79036813 (*PTCH1*, OR_A_ = 0.42, p = 2.6 × 10^–3^).

**Conclusion:**

The results of this study, including a novel potential medulloblastoma risk loci at 18p11.23, are suggestive but need further validation in independent cohorts.

**Electronic supplementary material:**

The online version of this article (10.1007/s11060-020-03424-9) contains supplementary material, which is available to authorized users.

## Introduction

Medulloblastoma is the most common embryonal central nervous system malignancy in children. It is well known that a fraction of all cases is caused by germline mutations in *TP53* (underlying Li-Fraumeni syndrome), *APC* (underlying Turcot syndrome), or *PTCH1*/*PTCH2*/*SUFU* (underlying basal cell nevus/Gorlin, syndrome) [1, 2]. A recent study including 1022 medulloblastoma patients found that 6% of all cases had a germline mutation in *TP53*, *APC*, *PTCH1*, *SUFU*, or in two additional genes with presumed tumor suppressor function: *BRCA2* or *PALB2* [3]. Another recent study reported a novel medulloblastoma predisposition gene in *GPR161* [4]. The somatic genetic changes that occur in sporadic medulloblastoma tumors are also well-described, including alterations in *CCND2*, *CTNNB1*, *DDX3X*, *GLI2*, *SMARCA4*, *MYC*, *MYCN*, *PTCH1*, *TP53*, and *KMT2D* [5]. Although we know much about genetic aberrations in medulloblastoma tumors and the genetic syndromes that predispose to the disease, little is known about how common germline genetic variants (i.e. single nucleotide polymorphisms, SNPs) contribute to medulloblastoma susceptibility.

Prognosis for medulloblastoma patients is poor, with a ten-year survival rate of 63% [6]. As a consequence of the disease and intensive treatment, the children who survive have an increased risk of long-term neurocognitive dysfunction and secondary malignancies [7]. To improve treatment and prevention strategies for this devastating disease, a better understanding of medulloblastoma etiology is needed. We have conducted a genome-wide association study (GWAS) with the aim to identify genetic variants that are associated with medulloblastoma development in children and young adults. Identifying genetic variants that predispose to medulloblastoma development may provide new insights into the genetic pathways that contribute to the development of the disease and potential new targets for therapy.

## Results

To find germline genetic variants associated with medulloblastoma risk, we conducted a genome-wide scan of 244 medulloblastoma cases and 247 control subjects from Sweden and Denmark that fulfilled the inclusion criteria (Figure S1; Table S1). Tests of association with medulloblastoma risk were performed for 1,288,472 SNPs that passed quality control. The Q–Q plot and inflation factor ʎ indicated no significant effect on the results by population stratification (Figure S2). Thirteen genetic variants in six genomic loci were associated with increased medulloblastoma risk (p < 1 × 10^–5^), but none were statistically significant when applying a conservative p-value threshold to adjust for multiple testing (p < 5 × 10^–8^; Table 1). We were able to analyze 12 of these variants in a validation cohort consisting of 249 cases and 629 controls (Table S1). In the validation cohort, one genetic variant, rs78021424 (18p11.23, *PTPRM*), was associated with medulloblastoma risk with an OR in the same direction as in the discovery cohort (Table 1).

**Table 1:** Top SNPs from association analyses of 1,288,472 directly genotyped SNPs

SNP	Major/minor allele	Discovery				Validation		
		maf controls/cases	OR	95% CI	p-value	OR	p-value	loci (genes within 30,000 bp)
rs853362	A/G	0.142/0.262	2.06	1.51–2.83	6.49 × 10^–6^	1.19	0.2546	6p23 (*CD83)*
rs853372	G/A	0.142/0.26	2.05	1.49–2.82	9.18 × 10^–6^	1.13	0.4184	6p23 (*CD83)*
rs10266582	C/T	0.152/0.059	0.32	0.21–0.50	2.41 × 10^–7^	1.46	0.0302	7q21.11 (*MAGI2)*
rs17404544	T/C	0.063/0.143	2.58	1.70–3.93	9.05 × 10^–6^	1.33	0.3677	8p23.2 (*CSMD1)*
rs80012312	A/G	0.002/0.053	7.35	3.31–16.30	9.25 × 10^–7^	n.a	n.a	8q24.12
rs7077776	A/C	0.245/0.373	1.85	1.41–2.43	9.92 × 10^–6^	1.07	0.5842	10q26.2 (*DOCK1)*
rs11661715	A/G	0.036/0.109	3.83	2.28–6.43	3.67 × 10^–7^	1.04	0.8652	18p11.23 (*PTPRM)*
rs11873445	C/T	0.04/0.119	3.91	2.37–6.45	9.55 × 10^–8^	1.15	0.5116	18p11.23 (*PTPRM)*
rs12185387	A/G	0.043/0.121	3.63	2.23–5.90	2.24 × 10^–7^	1.04	0.8364	18p11.23 (*PTPRM)*
rs12956144	T/C	0.04/0.117	3.81	2.30–6.30	1.87 × 10^–7^	1.03	0.8908	18p11.23 (*PTPRM)*
rs78021424	C/T	0.04/0.115	3.77	2.27–6.25	2.81 × 10^–7^	1.59	0.0209	18p11.23 (*PTPRM)*
rs1468707	G/A	0.043/0.117	3.69	2.23–6.09	3.29 × 10^–7^	1.03	0.8975	18p11.23 (*PTPRM)*
rs1942957	A/G	0.043/0.117	3.69	2.23–6.09	3.29 × 10^–7^	1.05	0.8095	18p11.23 (*PTPRM)*

**Fig. 1:**
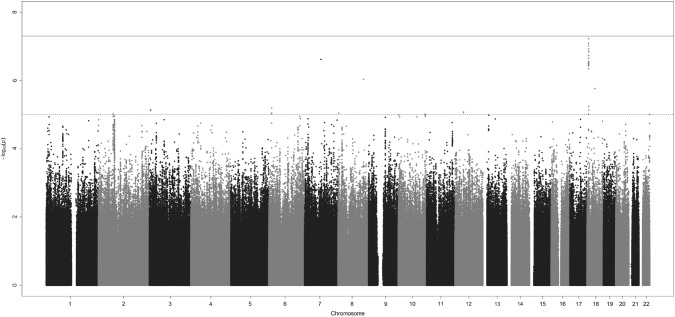
Manhattan plot. P-values for the association between 9,204,561 genetic variants and medulloblastoma risk. Both genotyped and imputed SNPs are included. Solid line indicates genome-wide statistical significance (p = 5 × 10^–8^). Dashed line indicates p = 1 × 10^–5^

In a search for SNPs with even stronger associations at the 18p11.23 locus, and to find additional interesting regions, we imputed SNPs in the discovery dataset and performed association analyses of an additional 7,916,089 SNPs (Fig. 1). Forty-six imputed SNPs in eight genomic loci were associated with medulloblastoma risk (p < 1 × 10^–5^; Table S2). These associations were not, however, statistically significant after adjusting for multiple testing. The SNP with the strongest association in the 18p11.23 (*PTPRM*) locus was rs185966860 (OR_per A allele_ = 4.01, 95% CI 2.43–6.63, p  = 5.97 × 10^–8^).

In addition to genome-wide analyses, we were specifically interested in seven genes, namely: *APC*, *BRCA2*, *PALB2*, *PTCH1*, *SUFU*, *TP53*, and *GPR161* [3, 4]. Within these seven candidate genes, the strongest evidence for association was found for rs201458864, located within *PALB2* (OR_per T allele_ = 3.76, 95% CI 1.83–7.75, p = 3.2 × 10^–4^) and rs79036813, located within *PTCH1* (OR_per A allele_ = 0.42, 95% CI 0.24–0.74, p = 2.6 × 10^–3^) (Figure S3).

## Discussion

In this first GWAS of medulloblastoma, we found a potential medulloblastoma risk locus at 18p11.23. Medulloblastoma is a rare disease, which makes it challenging to collect enough samples for adequate statistical power, especially for a GWAS. Compared to other epidemiologic studies of medulloblastoma, the number of cases included in this study is large. However, in relation to the number of statistical tests performed, the number of cases is still small, and our study was not powered to detect associations with a small effect size. Although GWAS of adult cancers usually report associations with small effect sizes, studies of early onset malignancies have reported associations with larger effects [8]. Analogous with this, for the majority of associations with p < 1 × 10^–5^ in this study, effect sizes were large, and carriers of the risk allele had a more than two-fold increased risk. Our findings were not statistically significant when using the p value threshold p < 5 × 10^–8^ to correct for multiple comparisons. Although a stringent p-value threshold is required in GWAS to reduce the presence of false positive findings, strict Bonferroni correction may be considered overly conservative due to linkage disequilibrium between many genetic variants. Twelve variants with evidence for associations in the initial analyses were investigated in an additional cohort. One of these SNPs, located in 18p11.23 (*PTPRM*) showed suggestive evidence for an association with medulloblastoma risk also in the validation cohort. The *PTPRM* gene product is a receptor-type protein tyrosine phosphatase that mediates cell–cell adhesion. Altered expression, mutations, or aberrant methylation of *PTPRM* have been described in different malignancies, including glioblastoma [9]. The role of *PTPRM* in medulloblastoma is, to our knowledge, unknown, but it is interesting to note that the *PTPRM* protein has been shown to interact with beta-catenin [10]. Beta-catenin is a central part of the Wnt signaling pathway and is encoded by the gene *CTNNB1*, which is frequently mutated in WNT medulloblastoma [5]. However, only about 10% of all medulloblastoma tumors belong to the WNT subgroup [5], and this subgroup is therefore represented by few patients in the study cohort. Investigation of imputed variants across the genome indicated the presence of additional variants associated with medulloblastoma risk in the 18p11.23 locus and variants in five additional genetic regions that remain to be validated in an independent cohort.

Germline mutations in *APC*, *BRCA2*, *PALB2*, *PTCH1*, *SUFU*, and *TP53* occur in up to 6% of all medulloblastoma cases [3]**.** Another potential medulloblastoma predisposing mutation has been reported in the gene *GPR161* [4]. In candidate gene analysis restricted to these seven genes, we observed associations between genetic variants in *PALB2* and *PTCH1* and medulloblastoma risk. Pathogenic genetic variants in *PALB2* have been associated with increased risk of medulloblastoma as well as breast cancer [3, 11]. Genetic testing of *PALB2* has been suggested for clinical testing in breast cancer families and in specific subgroups of medulloblastoma based on clinical and molecular tumor characteristics [3, 12]. Germline mutations in *PTCH1* give rise to basal cell nevus (Gorlin) syndrome, which comes with an increased risk of different malignancies, including basal cell carcinoma and medulloblastoma. In the present study, we investigated common germline genetic variants (minor allele frequency > 1%), and we could not assess the rare germline mutations in *PALB2* and *PTCH1* reported by Waszak et al. [3].

Medulloblastoma tumors comprise four or more molecular subgroups [5]. The cases in our discovery sets were diagnosed during a period when these molecular subgroups of medulloblastoma were not established. In the present study, tissue samples are not possible to obtain, and molecular subgroups cannot be taken into consideration in the analyses, which is a limitation of the study. In GWAS of glioma, which is also a heterogeneous group of brain tumors, we and others have shown that many established risk loci are specific for certain subtypes [13, 14]. However, even in early GWAS of glioma, in which all glioma were included, we found several genetic variants that were associated with an increased risk of all glioma, irrespective of molecular subtype [15]. Another potential limitation of the study is the inclusion of study subjects from six different countries in the validation phase, whereas patients and control subjects in the discovery phase were born in either Sweden or Denmark.

An advantage of the study is that, although cases were retrospectively identified, their blood samples were collected prior to disease diagnosis. With this study design, we avoided survival bias, which can be a problem in a case–control study of an aggressive disease, where mortal cases would be underrepresented. On the contrary, we may have an underrepresentation of less aggressive medulloblastoma, since a subset of surviving cases chose not to participate in the study.

In summary, we have identified 11 loci that may be associated with medulloblastoma development in children and young adults, including the 18p11.23 (*PTPRM*) loci that was validated in a separate cohort. None of the observed associations were, however, statistically significant after conservative correction for multiple testing, and to know the relevance of these loci in medulloblastoma etiology, replication in independent cohorts is needed. If these associations proves robust in independent validations, it is a step towards enhanced understanding of medulloblastoma etiology, which in turn may enable development of improved treatment and prevention strategies. For sufficient power of future studies of genetic variants in medulloblastoma, broad international collaborations are required.

## Materials and methods

### Study subjects

Medulloblastoma cases diagnosed between 1975 and 2008, under the age of 25, were identified from the national cancer registries in Sweden (n = 136) and Denmark (n = 128) [16] (Table S1). Dried blood spot samples were collected from the Swedish Phenylketonuria Screening Registry [17] and the Danish Newborn Screening Biobank, which are national biobanks containing dried blood spot samples from newborns. For each medulloblastoma case, one control subject was identified among samples that were physically located close to the case sample in the biobanks. Control subjects were matched by date of birth (Swedish and Danish controls) and sex (Danish controls only).

In Sweden, the study was approved by the Data Inspection Board and the Regional Ethical Review Board. All living Swedish subjects provided informed consent. The Regional Ethical Review Board approved the use of samples from deceased Swedish cases without informed consent from close relatives. In Denmark, the study was approved by the Research Ethics committee of the Capital Region (Copenhagen), the Danish Data Protection Agency, and by the Danish Newborn Screening Biobank Steering Committee. According to Danish law, the regional Ethics Committee can grant exemption from obtaining informed consent for research projects on biobank samples under certain circumstances [18]. For this study, such an exemption was granted.

The validation study included 249 cases and 629 controls originally recruited to four different studies: (1) Studies at Children’s Hospital Los Angeles and the USC Keck School of Medicine (CA, USA) [19], (2) a study conducted at Baylor College of Medicine in Houston (TX, USA), (3) a study conducted at the University of Medical Sciences in Poznan, Poland, and (4) the CEFALO study conducted in Denmark, Sweden, Norway, and Switzerland [20] (Table S1). Ethical approval and informed consent from validation study subjects were obtained at the respective study site.

### Genotyping and imputation

DNA extraction and genotyping have previously been described in detail [16]. In brief, DNA was extracted using the Extract-N-amp kit (Sigma-Aldrich) [21–23] and was whole-genome-amplified using the REPLIg kit (QIAGEN; Danish subjects) or the GenomePlex Single Cell Whole Genome Amplification kit (Sigma-Aldrich; Swedish subjects). Genotyping was performed using a high-density SNP-array (HumanOmni2.5–8 BeadChip, Illumina). Subjects were excluded if their call-rate was less than 97% or if technical issues were identified, for example conflicting information on reported sex versus X chromosome genotypes or the presence of unexpected duplicate samples. We also excluded subjects identified as outliers using principal component analysis [24, 25] (Figure S2). Based on these criteria, 20 cases and 17 controls were excluded (Figure S1). All subjects included in the association analyses were unrelated (PI-HAT < 0.2). SNPs were excluded based on call-rate (< 95%), minor allele frequency (MAF) (< 1%), and Hardy–Weinberg test (p < 1 × 10^–4^). We also excluded any A/T and C/G SNPs. Quality control was performed using PLINK (version 1.07, https://zzz.bwh.harvard.edu/plink/) [26]. Imputation was based on 1,288,472 SNPs that passed quality control in the Swedish and Danish datasets and was performed using IMPUTE2 and SHAPEIT2 software and data from the 1000 Genomes Project as reference [27–30]. Imputed SNPs with MAF < 1% or imputation info score < 0.8 were excluded from all subsequent analyses.

In the validation phase of the study, we used the Sequenom iPLEX Gold platform when genotyping all subjects, except for control subjects from the study conducted at Children’s Hospital Los Angeles and the USC Keck School of Medicine. These subjects were genotyped using Illumina BeadChips, and SNPs that were not represented on the arrays were imputed using MACH v1.0 and the HapMap phase 2 release 21 consensus CEU or CEU + ASN haplotypes as reference.

### Selection of SNPs

The genes *APC*, *BRCA2*, *PALB2*, *PTCH1*, *SUFU*, *TP53*, and *GPR161* were selected for investigation using a candidate gene approach. The selection was based on two recent studies that found germline mutations in one of these genes in 6% of all medulloblastoma cases [3, 4]. The 1446 SNPs located within these genes include directly genotyped as well as imputed SNPs. We have previously reported the association between genotyped variants in *PTCH1* and *TP53* and medulloblastoma risk based on the same study population [16].

### Statistical methods

Association between genetic variants and medulloblastoma risk was assessed using a frequentist test under an additive model and the score method using SNPTEST v2.5.2 [31]. Analyses were adjusted for sex and five principal components. Principal component analyses were conducted using EIGENSOFT version 6.1.4 (https://www.hsph.harvard.edu/alkes-price/software/) [24, 25].

In the validation phase of the study, logistic regression analysis was preformed separately in two subsets of validation study subjects. Subset 1 included subjects from Children’s Hospital Los Angeles and the USC Keck School of Medicine, and subset 2 included all other validation study subjects. The results from these two subsets were then combined using fixed-effect model meta-analysis.

In genome-wide (agnostic) analyses, p < 5 × 10^–8^ was considered statistically significant. For candidate gene analyses, p < 0.007 was considered statistically significant, corresponding to Bonferroni correction for testing seven independent loci.

## Electronic supplementary material

Below is the link to the electronic supplementary material.

Figure S1, Subject Inclusion; Figure S2, PCA and Q-Q plots; Figure S3, Candidate Genes; Table S1, Study Subjects; Table S2, Genotyped and imputed genetic variants associated with medulloblastoma risk (p < 1 × 10^−5^) (PDF 921 kb)

## Data Availability

The datasets generated and/or analysed during the current study are not publicly available due to legal restrictions but are available from the corresponding author on reasonable request for researchers who meet the criteria for access to confidential data.
