# Supplementary material for: A genome-wide association study on medulloblastoma
Source: J Neurooncol. 2020 Feb 13;147(2):309–15. doi: 10.1007/s11060-020-03424-9 (PMC7136185; doi:10.1007/s11060-020-03424-9)
Supplement: Supplementary file 1 — Figure S1, Subject Inclusion; Figure S2, PCA and Q-Q plots; Figure S3, Candidate Genes; Table S1, Study Subjects; Table S2, Genotyped and imputed genetic variants associated with medulloblastoma risk (p < 1 × 10^−5^) (PDF 921 kb) [file 11060_2020_3424_MOESM1_ESM.pdf]

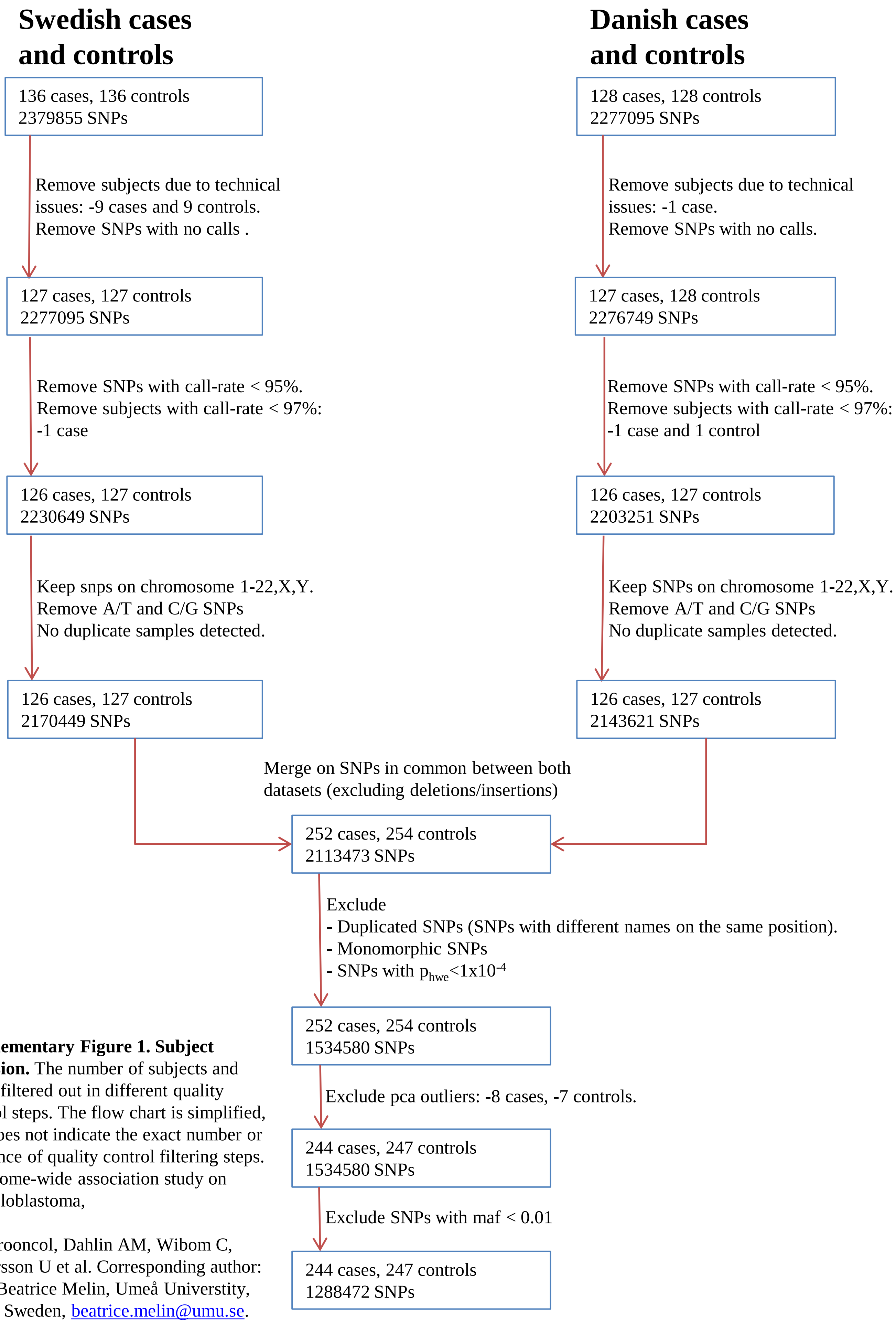

Supplementary Figure 1. Subject inclusion. The number of subjects and SNPs filtered out in different quality control steps. The flow chart is simplified, and does not indicate the exact number or sequence of quality control filtering steps. A genome-wide association study on medulloblastoma,

J Neurooncol, Dahlin AM, Wibom C, Andersson U et al. Corresponding author: Prof. Beatrice Melin, Umeå Universtity, Umeå Sweden, beatrice.melin@umu.se.

Supplementary Figure 2. Q-Q plot (A) and PCA plots before (B) and after (C) exclusion of outlier individuals. In (B) and (C), subjects are colored according to case/control status and country. Orange, Danish controls; red, Danish cases; light blue, Swedish controls; Blue, Swedish cases.

A genome-wide association study on medulloblastoma, J Neurooncol, Dahlin AM, Wibom C, Andersson U et al.
Corresponding author: Prof. Beatrice Melin, Umeå Universtity, Umeå Sweden, beatrice.melin@umu.se.

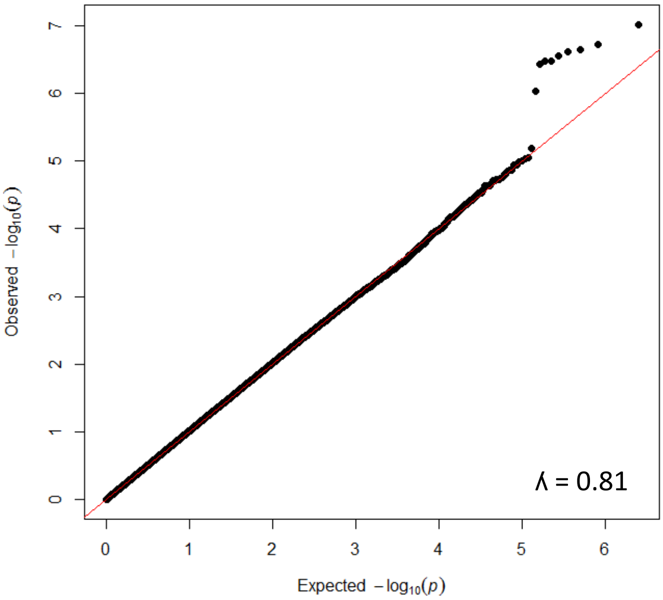

A

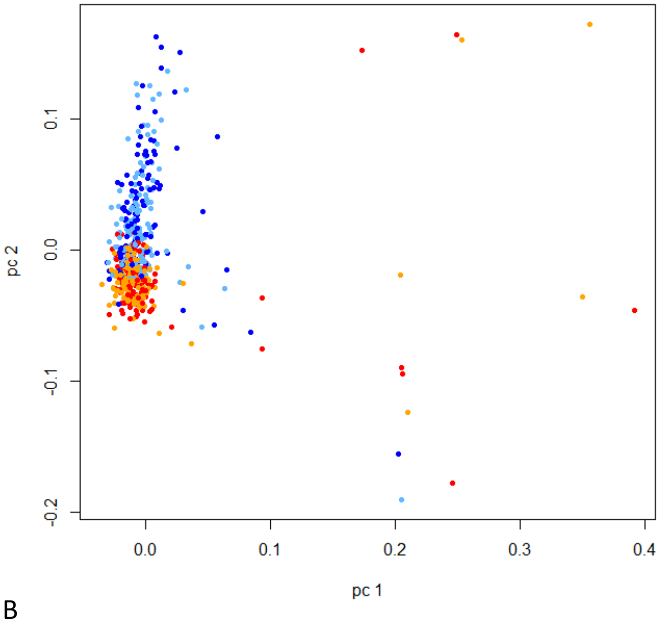

B

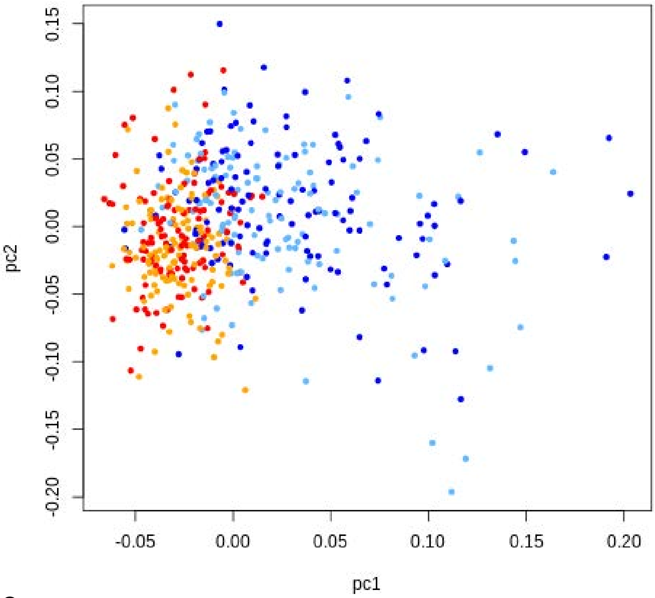

C

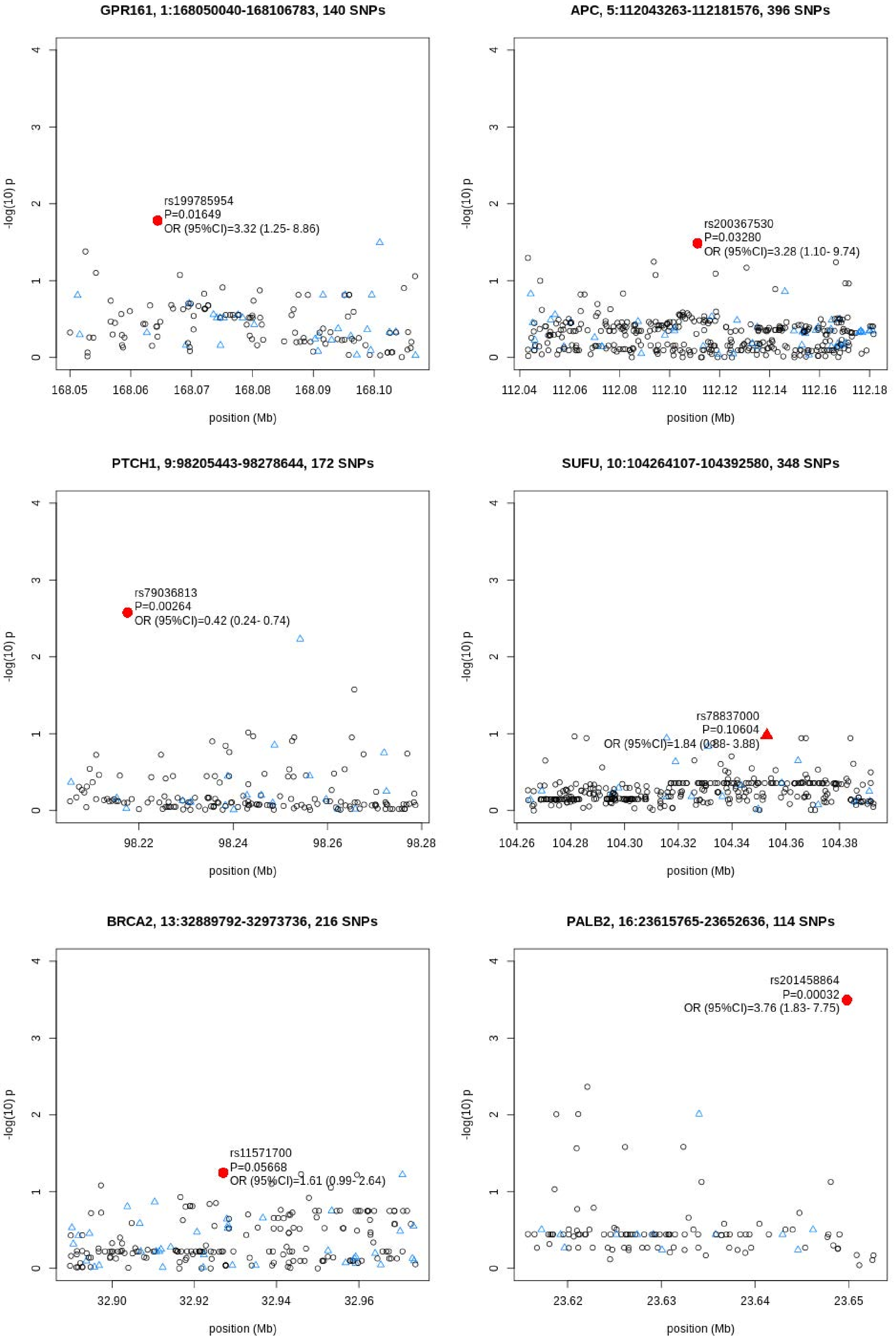

GPR161, 1:168050040-168106783, 140 SNPs
rs199785954
P=0.01649
OR (95%CI)=3.32 (1.25- 8.86)
-log(10) p
position (Mb)
APC, 5:112043263-112181576, 396 SNPs
rs200367530
P=0.03280
OR (95%CI)=3.28 (1.10- 9.74)
-log(10) p
position (Mb)
PTCH1, 9:98205443-98278644, 172 SNPs
rs79036813
P=0.00264
OR (95%CI)=0.42 (0.24- 0.74)
-log(10) p
position (Mb)
SUFU, 10:104264107-104392580, 348 SNPs
rs78837000
P=0.10604
OR (95%CI)=1.84 (0.88- 3.88)
-log(10) p
position (Mb)
BRCA2, 13:32889792-32973736, 216 SNPs
rs11571700
P=0.05668
OR (95%CI)=1.61 (0.99- 2.64)
-log(10) p
position (Mb)
PALB2, 16:23615765-23652636, 114 SNPs
rs201458864
P=0.00032
OR (95%CI)=3.76 (1.83- 7.75)
-log(10) p
position (Mb)

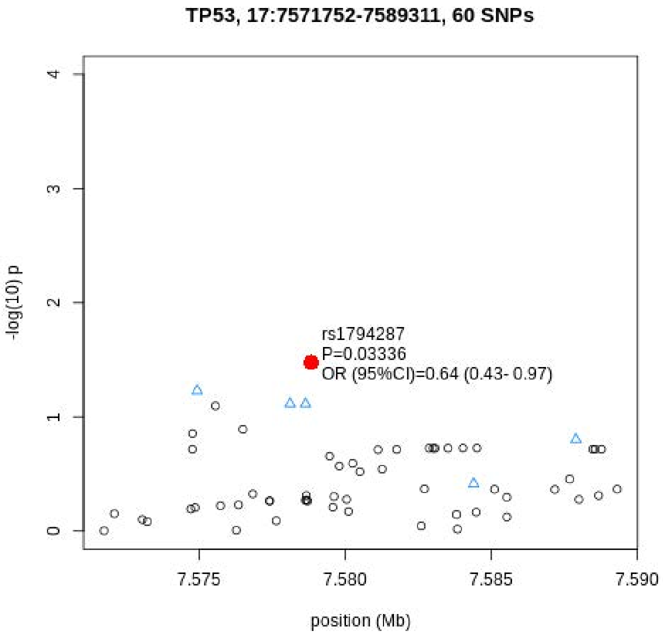

Supplementary Figure 3. P-values for association between risk of medulloblastoma and genetic variants in genes in which germline mutations are frequent in medulloblastoma patients. A filled red symbol marks the SNP with the lowest p-value in the region. The name, p-value, odds ratio, and 95% confidence interval of the SNP with the lowest p-value are given. Genotyped SNPs are marked with blue triangles and imputed SNPs are marked with black circles. P-values are not adjusted for multiple testing.

A genome-wide association study on medulloblastoma, J Neurooncol, Dahlin AM, Wibom C, Andersson U et al. Corresponding author: Prof. Beatrice Melin, Umeå Universtity, Umeå Sweden, beatrice.melin@umu.se.

A genome-wide association study on medulloblastoma, J Neurooncol,
Dahlin AM, Wibom C, Andersson U et al. Corresponding author: Prof.
Beatrice Melin, Umeå Universtity, Umeå Sweden, beatrice.melin@umu.se.

Supplementary Table 1. Study subjects.

	**Medulloblastoma cases**		**Control subjects**	
	**N (males/ females)**	**Samples**	**N (males/ females)**	**Samples**
**Discovery**				
Swedish subjects [1]	124 (77/47)	Neonatal dried blood spot samples from cases born 1975-2002, diagnosed before the age of 25, identified from the national cancer register.	124 (61/64)	Neonatal dried blood spot samples from control subjects matched to cases by year of birth.
Danish subjects [1]	120 (68/52)	Neonatal dried blood spot samples from cases born 1982- 2008, diagnosed before the age of 20, identified from the national cancer register.	122 (68/54)	Neonatal dried blood spot samples from control subjects matched to cases by year of birth and sex.
**Validation**				
CEFALO (Denmark, Sweden, Norway, and Switzerland) [2]	31 (19/12)	Saliva samples from cases recruited to CEFALO (ref [2]), diagnosed 2004-2008.[a] Age range, 7-19 years.	89 (52/37)	Saliva samples from control subjects recruited to CEFALO (ref [2]). Matched to cases by age, sex, and region.
Baylor College of Medicine, TX, USA	124 (89/35)	Blood samples from cases treated at Texas Children's Cancer Center, 1987-2011. Age range, 0-18 years.	139 (86/53)	Saliva samples from healthy subjects from the Houston area identified at a general pediatrics clinic associated with Texas Children's Hospital. Controls presented for a well-child visit or sports physical (subjects presenting for an illness were not included). Matched to cases by age, gender, and race/ethnicity.
Poznan University of Medical Sciences, Poznan, Poland	21 (10/11)	Blood samples from cases treated at the Dept. of Pediatric Oncology, Hematology and Transplantation, Poznan University of Medical Sciences, 2003-2012. Age range, 0-18 years.	89 (48/41)	Blood samples from blood donors from the region of Wielkopolska, Poland, collected 2007-2008. Blood donor history did not indicate a history of chronic diseases, including cancer. Age range, 16-65 years.
Childrens Hospital Los Angeles, CA, USA	73 (40/33)	Blood or Peripheral Blood Mononuclear Cell samples from cases treated at Childrens Hospital Los Angeles. Age range, 0-21 years.	312 (180/132)	Blood samples from control subjects originally recruited to The Children's Health Study out of Keck School of Medicine at the University of Southern California [3]. All control subjects were Hispanic white or non-Hispanic white. Matched to cases by gender and ethnicity.
**Total**				
Discovery	244 (145/99)		247 (129/118)	
Validation	249 (158/91)		629 (366/263)	

[a]CEFALO and the Danish discovery study overlap in their recruitment of subjects. We excluded 10 Danish CEFALO cases that were potentially included in both studies.

A genome-wide association study on medulloblastoma, J Neurooncol, Dahlin AM, Wibom C, Andersson U et al. Corresponding author: Prof. Beatrice Melin, Umeå Universtity, Umeå Sweden, beatrice.melin@umu.se.

1. Dahlin, A.M.; Hollegaard, M.V.; Wibom, C.; Andersson, U.; Hougaard, D.M.; Deltour, I.; Hjalmars, U.; Melin, B. *J Neurooncol* **2015**, *125*, 75-78, doi:10.1007/s11060-015-1891-1.
2. Aydin, D.; Feychting, M.; Schuz, J.; Tynes, T.; Andersen, T.V.; Schmidt, L.S.; Poulsen, A.H.; Johansen, C.; Prochazka, M.; Lannering, B., et al. *J Natl Cancer Inst* **2011**, *103*, 1264-1276, doi:10.1093/jnci/djr244.
3. Torgerson, D.G.; Ampleford, E.J.; Chiu, G.Y.; Gauderman, W.J.; Gignoux, C.R.; Graves, P.E.; Himes, B.E.; Levin, A.M.; Mathias, R.A.; Hancock, D.B., et al. *Nat Genet* **2011**, *43*, 887-892, doi:10.1038/ng.888.

Supplementary Table 2. Genotyped and imputed genetic variants associated with and medulloblastoma risk ($p < 1 \times 10^{-5}$).

SNP	chromosome:position (GRCh37/hg19)	major/minor allele	maf controls/cases	OR[a]	95% CI	p-value	genes within 30000 bp	loci	genotyped /imputed	imputation info score
rs201616177	2:71051416	CCACCATCT/C	0.423/0.298	0.54	0.41- 0.71	9.57E-06	CLEC4F,CD207	2p13.3	imputed	0.983
rs72907985	2:71051431	C/T	0.423/0.298	0.54	0.41- 0.71	9.51E-06	CLEC4F,CD207	2p13.3	imputed	0.983
rs72907986	2:71051434	A/G	0.423/0.298	0.54	0.41- 0.71	9.51E-06	CLEC4F,CD207	2p13.3	imputed	0.983
rs201563714	3:5937331	CT/C	0.202/0.317	2.07	1.51- 2.85	7.38E-06		3p26.1	imputed	0.832
rs853362	6:14138427	A/G	0.142/0.262	2.06	1.51- 2.83	6.49E-06	CD83	6p23	genotyped	1
rs853366	6:14141151	G/C	0.142/0.26	2.05	1.49- 2.82	9.37E-06	CD83	6p23	imputed	0.999
rs750749	6:14142097	T/C	0.142/0.26	2.05	1.49- 2.82	9.29E-06	CD83	6p23	imputed	1
rs853370	6:14142674	G/A	0.142/0.26	2.05	1.49- 2.82	9.19E-06	CD83	6p23	imputed	0.999
rs853371	6:14142944	A/G	0.142/0.26	2.05	1.49- 2.82	9.25E-06	CD83	6p23	imputed	1
rs11435395	6:14143756	T/TA	0.142/0.26	2.05	1.49- 2.82	9.19E-06	CD83	6p23	imputed	1
rs853372	6:14143961	G/A	0.142/0.26	2.05	1.49- 2.82	9.18E-06	CD83	6p23	genotyped	1
rs866469	6:14144022	G/A	0.142/0.26	2.05	1.49- 2.82	9.13E-06	CD83	6p23	imputed	1
rs853373	6:14144105	A/G	0.142/0.26	2.05	1.49- 2.82	9.07E-06	CD83	6p23	imputed	0.999
rs944487	6:14144212	G/A	0.142/0.26	2.06	1.50- 2.83	9.00E-06	CD83	6p23	imputed	0.999
rs10266582	7:78066878	C/T	0.152/0.059	0.32	0.21- 0.50	2.41E-07	MAGI2	7q21.11	genotyped	1
rs17404544	8:3964539	T/C	0.063/0.143	2.58	1.70- 3.93	9.05E-06	CSMD1	8p23.2	genotyped	1
rs80012312	8:122362199	A/G	0.002/0.053	7.35	3.31-16.30	9.25E-07		8q24.12	genotyped	1
rs11016880	10:129038744	A/G	0.244/0.368	1.88	1.42- 2.48	9.68E-06	DOCK1	10q26.2	imputed	0.992
rs7077776	10:129041541	A/C	0.245/0.373	1.85	1.41- 2.43	9.92E-06	DOCK1	10q26.2	genotyped	1
rs1596519	12:41198114	A/T	0.408/0.277	0.54	0.41- 0.71	8.40E-06	CNTN1	12q12	imputed	0.988
rs1596518	12:41198144	T/C	0.408/0.277	0.54	0.41- 0.71	8.59E-06	CNTN1	12q12	imputed	0.987
rs8088882	18:7930046	A/G	0.037/0.109	3.85	2.29- 6.47	3.71E-07	PTPRM	18p11.23	imputed	0.991
rs8093794	18:7931441	C/T	0.037/0.109	3.85	2.29- 6.46	3.67E-07	PTPRM	18p11.23	imputed	0.993
rs11661715	18:7947184	A/G	0.036/0.109	3.83	2.28- 6.43	3.67E-07	PTPRM	18p11.23	genotyped	1
rs80327499	18:7948407	G/A	0.036/0.109	3.83	2.28- 6.43	3.67E-07	PTPRM	18p11.23	imputed	1
rs144957179	18:7960790	CAT/C	0.036/0.108	3.85	2.29- 6.47	3.55E-07	PTPRM	18p11.23	imputed	0.996
rs2044220	18:7964648	C/G	0.036/0.108	3.95	2.34- 6.68	2.76E-07	PTPRM	18p11.23	imputed	0.985
rs11664885	18:7977234	C/T	0.042/0.119	3.85	2.33- 6.37	1.43E-07	PTPRM	18p11.23	imputed	0.988
rs11081349	18:7977468	T/C	0.042/0.117	3.84	2.31- 6.36	1.87E-07	PTPRM	18p11.23	imputed	0.989
rs17476082	18:7980686	G/C	0.042/0.119	3.85	2.33- 6.36	1.39E-07	PTPRM	18p11.23	imputed	0.994
rs78361801	18:7982545	T/C	0.041/0.119	3.88	2.35- 6.40	1.18E-07	PTPRM	18p11.23	imputed	0.996
rs111748772	18:7984466	T/C	0.041/0.118	3.99	2.41- 6.61	8.08E-08	PTPRM	18p11.23	imputed	0.99
rs185966860	18:7984976	T/A	0.041/0.12	4.01	2.43- 6.63	5.97E-08	PTPRM	18p11.23	imputed	0.991
rs11873208	18:7997263	G/C	0.04/0.119	3.91	2.37- 6.45	9.55E-08	PTPRM	18p11.23	imputed	1
rs11873445	18:7997409	C/T	0.04/0.119	3.91	2.37- 6.45	9.55E-08	PTPRM	18p11.23	genotyped	1
rs12185387	18:8001853	A/G	0.043/0.121	3.63	2.23- 5.90	2.24E-07	PTPRM	18p11.23	genotyped	1
rs12956144	18:8004324	T/C	0.04/0.117	3.81	2.30- 6.30	1.87E-07	PTPRM	18p11.23	genotyped	1
rs76087235	18:8007530	G/A	0.041/0.115	3.77	2.27- 6.26	2.77E-07	PTPRM	18p11.23	imputed	0.998
rs11660294	18:8009675	C/T	0.042/0.115	3.67	2.22- 6.09	4.52E-07	PTPRM	18p11.23	imputed	0.996
rs6506543	18:8012953	C/G	0.041/0.115	3.72	2.24- 6.17	3.71E-07	PTPRM	18p11.23	imputed	0.995
rs11081353	18:8014251	C/G	0.04/0.115	3.77	2.27- 6.25	2.81E-07	PTPRM	18p11.23	imputed	1
rs78021424	18:8014429	C/T	0.04/0.115	3.77	2.27- 6.25	2.81E-07	PTPRM	18p11.23	genotyped	1
rs13381562	18:8019465	A/G	0.057/0.133	2.72	1.75- 4.24	9.57E-06	PTPRM	18p11.23	imputed	0.995
rs4461180	18:8029609	T/C	0.041/0.115	3.77	2.27- 6.24	2.77E-07	PTPRM	18p11.23	imputed	0.994
rs143219893	18:8038980	G/GT	0.042/0.116	3.76	2.27- 6.23	2.92E-07	PTPRM	18p11.23	imputed	0.989
rs58457935	18:8048584	A/T	0.054/0.125	2.93	1.84- 4.67	5.79E-06	PTPRM	18p11.23	imputed	0.995
rs11876715	18:8052364	C/G	0.042/0.116	3.72	2.25- 6.14	3.10E-07	PTPRM	18p11.23	imputed	0.996
rs77961746	18:8052812	T/G	0.042/0.116	3.71	2.25- 6.14	3.10E-07	PTPRM	18p11.23	imputed	0.996
rs77170547	18:8057348	C/T	0.042/0.116	3.7	2.24- 6.12	3.25E-07	PTPRM	18p11.23	imputed	0.996
rs1468707	18:8067216	G/A	0.043/0.117	3.69	2.23- 6.09	3.29E-07	PTPRM	18p11.23	genotyped	1
rs11660124	18:8067584	C/T	0.043/0.117	3.69	2.23- 6.09	3.29E-07	PTPRM	18p11.23	imputed	1
rs149503536	18:8068912	A/C	0.042/0.117	3.69	2.24- 6.09	3.30E-07	PTPRM	18p11.23	imputed	0.999
rs112654291	18:8068951	C/A	0.055/0.125	2.88	1.81- 4.58	7.29E-06	PTPRM	18p11.23	imputed	0.996
rs17396187	18:8073421	T/C	0.043/0.117	3.69	2.23- 6.09	3.29E-07	PTPRM	18p11.23	imputed	1
rs77910508	18:8077849	T/A	0.043/0.117	3.69	2.23- 6.09	3.29E-07	PTPRM	18p11.23	imputed	1
rs1942957	18:8094787	A/G	0.043/0.117	3.69	2.23- 6.09	3.29E-07	PTPRM	18p11.23	genotyped	1
rs75623572	18:8095690	C/A	0.043/0.117	3.69	2.24- 6.10	3.36E-07	PTPRM	18p11.23	imputed	0.996
rs34912034	18:37572523	T/TAC	0.558/0.423	0.49	0.37- 0.66	1.71E-06		18q12.3	imputed	0.827
rs2341366	22:50631146	A/G	0.116/0.219	2.17	1.54- 3.07	9.93E-06	PANX2,TRABD,SELENOO, TUBGCP6	22q13.33	imputed	0.975

[a] OR for the minor allele, calculated using the major allele as reference

A genome-wide association study on medulloblastoma, J Neurooncol, Dahlin AM, Wibom C, Andersson U et al.

Corresponding author: Prof. Beatrice Melin, Umeå Universtity, Umeå Sweden, beatrice.melin@umu.se.
